# PLM-ARG: antibiotic resistance gene identification using a pretrained protein language model

**DOI:** 10.1093/bioinformatics/btad690

**Published:** 2023-11-23

**Authors:** Jun Wu, Jian Ouyang, Haipeng Qin, Jiajia Zhou, Ruth Roberts, Rania Siam, Lan Wang, Weida Tong, Zhichao Liu, Tieliu Shi

**Affiliations:** Center for Bioinformatics and Computational Biology, and The Institute of Biomedical Sciences, School of Life Sciences, East China Normal University, Shanghai 200241, China; Center for Bioinformatics and Computational Biology, and The Institute of Biomedical Sciences, School of Life Sciences, East China Normal University, Shanghai 200241, China; Center for Bioinformatics and Computational Biology, and The Institute of Biomedical Sciences, School of Life Sciences, East China Normal University, Shanghai 200241, China; Center for Bioinformatics and Computational Biology, and The Institute of Biomedical Sciences, School of Life Sciences, East China Normal University, Shanghai 200241, China; ApconiX Ltd, Alderley Park, Alderley Edge SK10 4TG, United Kingdom; University of Birmingham, Birmingham B15 2TT, United Kingdom; Biology Department, School of Sciences and Engineering, The American University in Cairo, New Cairo 11835, Egypt; College of Architecture and Urban Planning, Tongji University, Shanghai 200092, China; National Center for Toxicological Research, Food and Drug Administration, Jefferson, AR 72079, United States; Nonclinical Drug Safety, Boehringer Ingelheim Pharmaceuticals, Inc, Ridgefield, CT 06877, United States; Center for Bioinformatics and Computational Biology, and The Institute of Biomedical Sciences, School of Life Sciences, East China Normal University, Shanghai 200241, China; School of Statistics, Key Laboratory of Advanced Theory and Application in Statistics and Data Science-MOE, East China Normal University, Shanghai 200062, China

## Abstract

**Motivation:**

Antibiotic resistance presents a formidable global challenge to public health and the environment. While considerable endeavors have been dedicated to identify antibiotic resistance genes (ARGs) for assessing the threat of antibiotic resistance, recent extensive investigations using metagenomic and metatranscriptomic approaches have unveiled a noteworthy concern. A significant fraction of proteins defies annotation through conventional sequence similarity-based methods, an issue that extends to ARGs, potentially leading to their under-recognition due to dissimilarities at the sequence level.

**Results:**

Herein, we proposed an Artificial Intelligence-powered ARG identification framework using a pretrained large protein language model, enabling ARG identification and resistance category classification simultaneously. The proposed PLM-ARG was developed based on the most comprehensive ARG and related resistance category information (>28K ARGs and associated 29 resistance categories), yielding Matthew’s correlation coefficients (MCCs) of 0.983 ± 0.001 by using a 5-fold cross-validation strategy. Furthermore, the PLM-ARG model was verified using an independent validation set and achieved an MCC of 0.838, outperforming other publicly available ARG prediction tools with an improvement range of 51.8%–107.9%. Moreover, the utility of the proposed PLM-ARG model was demonstrated by annotating resistance in the UniProt database and evaluating the impact of ARGs on the Earth's environmental microbiota.

**Availability and implementation:**

PLM-ARG is available for academic purposes at https://github.com/Junwu302/PLM-ARG, and a user-friendly webserver (http://www.unimd.org/PLM-ARG) is also provided.

## 1 Introduction

Antibiotic resistance is one of the biggest threats to global public health, food safety and security, and environmental sustainability (Dadgostar 2019, Murray *et al.* 2022). Antibiotic resistance leads to more extended hospital stays, higher medical costs, and increased mortality (Thorpe *et al.* 2018). According to the 2019 Antibiotic Resistance Threats Report released by the Centers for Disease Control and Prevention, >2.8 million antibiotic-resistant infections occur in the United States, resulting in over 35 000 people dying per year (https://www.cdc.gov/drugresistance/biggest-threats.html). Global efforts have been initiated to combat the escalating threat of antibiotic resistance, such as the Global Antimicrobial Resistance Surveillance System (Mendelson and Matsoso 2015, World Health Organization 2018), the Global Antibiotic Research and Development Partnership (Drugs for Neglected Diseases Initiative 2016), and the Interagency Coordination Group on Antimicrobial Resistance (Rochford *et al.* 2018). One focus of these consortium efforts is to develop a robust and efficient preclinical tool to predict antibiotic resistance. Notably, governmental agencies like the US Food and Drug Administration (FDA) are actively endorsing the One Health Initiative, which underscores the interconnected nature of health and environmental issues, specifically antibiotic resistance, and advocates for a comprehensive approach instead of a fragmented one (https://www.fda.gov/science-research/focus-areas-regulatory-science-report/cross-cutting-topics-one-health-initiative).

The horizontal transfer (HGT) of ARGs allows bacteria to exchange genetic information among different species, contributing to antibiotic resistance (Cao *et al.* 2021, Ellabaan *et al.* 2021, Wang *et al.* 2023). Therefore, identifying and quantifying ARGs has become one of the most effective ways to monitor antibiotic resistance. There are two main approaches for ARG identification, including sequence-based assembly/alignment (McArthur *et al.* 2013, Buchfink *et al.* 2015, Jia *et al.* 2017, Alcock *et al.* 2020) and machine learning techniques (Gibson *et al.* 2015, Arango-Argoty *et al.* 2018, Moradigaravand *et al.* 2018, Ruppé *et al.* 2019, Chowdhury *et al.* 2020). For sequence-based assembly/alignment, the queried sequence is directly compared to the known ARG reference database using alignment tools such as BLAST (Altschul *et al.* 1990), DIAMOND (Buchfink *et al.* 2015), and BWA (Li and Durbin 2009). Also, some modifications, such as local-based alignments, improve the performance of remote homolog identification (Feldgarden *et al.* 2019, Gibson *et al.* 2015). Although these methods performed well for highly conserved ARGs, they also generated a high false-negative rate for species-specific ARGs such as gram-negative bacteria (Chowdhury *et al.* 2019). In addition, several machine learning-based ARG identification models for identifying ARGs have been introduced, offering an alternative approach based on features representing characteristics of ARGs (Gibson *et al.* 2015, Arango-Argoty *et al.* 2018, Moradigaravand *et al.* 2018, Ruppé *et al.* 2019, Chowdhury *et al.* 2020). Nevertheless, these previously reported machine learning models were constructed using a limited set of genetic features derived from sequence data in specific bacterial species obtained from particular genomics technologies. This limitation restricts their broad applicability for ARG identification across diverse bacterial species.

The advent of metagenomic and metatranscriptomic technologies has unveiled a rich reservoir of proteins, significantly enhancing our understanding of microbial community functional diversity (Danko *et al.* 2021, Cai *et al.* 2022, Zhang *et al.* 2022). However, these studies have also highlighted a substantial challenge: a notable portion of proteins remains unannotated using homology-based or integrative information methods (Almeida *et al.* 2021, Wu *et al.* 2022). This issue extends to antibiotic resistance genes (ARGs), potentially causing them to remain unrecognized due to dissimilarities at the sequence level. Moreover, the application of 3D protein structures, believed to play a critical role in their biological function, is instrumental in ARG detection, with methods like pairwise comparative modeling (PCM) being used (Ruppe *et al.* 2019). Nevertheless, the availability of 3D protein structures of known ARGs is limited. Although, several outstanding protein structure prediction methods, e.g. AlphaFold2 (Jumper *et al.* 2021) and Rosetta (Du *et al.* 2021), have been developed, the confidence of the prediction results degraded when proteins lack homologous sequences, as the performance of these methods relies on the quality of multiple sequence alignment (Wang *et al.* 2022). In addition, the substantial computational resources required for structure prediction hinder their widespread application in large-scale metagenomic and metatranscriptomic studies for general research groups.

To uncover novel ARGs, elucidating the uncharacterized properties of proteins may be one of the most promising ways forward. Inspired by approaches proposed for natural language processing (NLP), the proteins could be represented as protein languages. Different protein-language models were applied to develop predictive methods for extracting complex sequence–structure–function relationships (Bepler and Berger 2021, Ofer *et al.* 2021, Unsal *et al.* 2022). Thus, a universal ARG prediction framework is urgently needed and can be provided by incorporating multi-omics data from different genomics technologies to offer a “one-stop” solution for ARG identification (Arango-Argoty *et al.* 2018, Li *et al.* 2021). Sequencing Quality Control Phase II (SEQC-II), led by the US FDA, aims to advise on best practices for sequence data analysis and to promote real-world applications of genomic technologies. As part of SEQC-II consortium efforts, we introduced a pioneering framework, PLM-ARG, meticulously crafted to discriminate ARGs, irrespective of their sequence similarity to well-established ARGs. This model harnesses the capabilities of ESM-1b, a publicly accessible protein language model comprising 650 million parameters, which has been trained on a dataset of approximately 250 million protein sequences (Rives *et al.* 2021).

**Figure 1.**
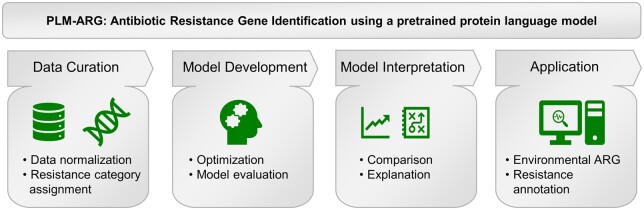
Overview of the proposed PLM-ARG framework.

This PLM-ARG framework consists of four components, including (i) ARG data curation and unification; (ii) protein language model-based ARG classification method development; (iii) model evaluation; and (iv) real-world applications (Fig. 1). In PLM-ARG, the most comprehensive ARG data were curated, unified, and manually assigned into different antibiotic resistance categories. In addition, we harnessed the publicly available ESM-1b model, accessible at https://github.com/facebookresearch/esm, to create embedding representations for the protein sequences. These embeddings were subsequently input into an XGBoost classifier, renowned for its implementation of gradient-boosting decision trees, and recognized for its outstanding performance across diverse classification challenges (Giacobbe 2020, Zhang *et al.* 2021, Lambert *et al.* 2022).

This integrated approach facilitates the concurrent identification of ARGs and the classification of resistance categories. Moreover, we demonstrated the utility of PLM-ARG by expanding the resistance annotation for ARGs in the Uniprot database and exploring the resistance diversity of the Earth's environmental microbiota.

## 2 Materials and methods

### 2.1 PLM-ARG model architecture

The proposed PLM-ARG model architecture encompasses a pretrained extensive protein language model to represent protein sequences. In addition, an inherent classification module is incorporated within this architecture, composed of two consecutive XGBoost models. These models are used for the specific tasks of ARG identification and the subsequent classification of these genes into distinct resistance categories. To establish a benchmark dataset for the development of the PLM-ARG model, we curated a comprehensive Antibiotic Resistance Gene database (PLM-ARGDB). This database was created by amalgamating and standardizing curated ARGs from five public ARG databases, followed by manual correction (see Supplementary Materials).

Briefly, we processed protein sequences through a transformer-based protein language model, ESM-1b, to generate embedding vectors. Each protein was then represented by a 1280-length numeric vector obtained by averaging the output from the 32nd layer of the ESM-1b model. Subsequently, we developed two XGBoost models using these protein representations for identifying ARGs and classifying their resistance categories. We opted for the XGBoost model due to its well-established prominence in the field of tabular data analysis. It is renowned for its outstanding performance, versatility, interpretability, and, notably, its reduced demand for hyperparameter tuning when compared to deep learning models (Shwartz-Ziv and Armon 2022). Five hyperparameters of the XGBoost model, specifically *n_estimators*, *max_depth*, *learning_rate*, *subsample*, and *colsample_bytree*, were meticulously optimized using the GridSearchCV procedure, involving 100 iterations of 3-fold cross-validation, as facilitated by Scikit-learn (Pedregosa *et al.* 2011). The model's performance was assessed based on the Area Under the Receiver Operating Characteristic curves (AUROC) values (see Supplementary Materials). Moreover, considering the computational requirements of ESM-1b model, including computational time and memory utilization, tend to rise proportionally with the length of input protein sequences, we conducted a systematic exploration of varying trimming lengths to ascertain an optimal trimming length that ensures both superior performance and computational efficiency (see Supplementary Materials).

### 2.2 Validation of PLM-ARG performance

Three state-of-the-art approaches for ARG classification, including RGI (a sequence alignment-based method) suggested by both CARD (Jia *et al.* 2017, Alcock *et al.* 2020) and MEGARes (version 5.2.0) (Lakin *et al.* 2017), Resfams (an HMM-based approach, version 1.2) (Gibson *et al.* 2015) and DeepARG (a deep-learning-based method, version v2) (Arango-Argoty *et al.* 2018), were selected as competitors for performance comparison. The threshold used to determine the queried proteins as ARGs was set as default for each method. In detail, DeepARG predicted proteins with a probability >0.8 as ARGs. Resfams considered proteins with an E-value <1e–10 were regarded as ARGs. For the RGI, proteins with perfect or strict hits were regarded as ARGs.

The comparison was 2-fold: (i) we implemented the three state-of-the-art ARG prediction methodologies with the curated data in PLM-ARGDB with a 5-fold cross-validation strategy. Then, we compared it to the performance of the PLM-ARG model. Briefly, the data was split into five sets. One set is taken for validation and the other four for training. We repeated the process for all five sets. (ii) We compared the optimized state-of-the-art ARG prediction models to the PLM-ARG model with the curated independent validation. Considering the curated independent validation set has no complete “ground-truth” information on the resistance categories, we only limited the comparison to the performance on ARG classification.

### 2.3 Performance metrics

Receiver operating characteristic (ROC) curves are used to evaluate model performance, generated by plotting sensitivity against specificity at different settings. For PLM-ARG and deepARG, we used the probability to calculate the AUROC. For the Resfam, we used the E-value to calculate AUROC. As the RGI used the “Loose,” “Strict,” and “Perfect” as criteria to determine the ARGs, we used the ratio of “Best_Hit_Bitscore” to “Pass_Bitscore” to calculate AUROC. Besides AUROC, we also evaluated the model using five other performance metrics, including accuracy, precision, recall, F1-score, and Matthews Correlation Coefficient (MCC) calculated as follows:


(1) Accuracy=TP+TNTP+FP+TN+FN

(2) Precision=TPTP+FP 

(3) Recall=TPTP+FN 

(4) F1=2×Precision×RecallPrecision+Recall 

(5) MCC=TP×TN-FP×FNTP+FPTP+FNTN+FPTN+FN 

TP, TN, FP, and FN denote true positive, true negative, false positive, and false negative. The model performance was calculated for the resistance category classification based on micro-averages for each performance metric.

### 2.4 Webserver construction

We utilized the Apache HTTP server as a web server, developed by PHP (Version: 7.0.12, https://www.php.net/) programming. Data interaction was implemented by HTML5, JavaScript, jQuery. All data in PLM-ARG are stored and managed in MySQL database (Version: 5.7.17, https://www.mysql.com/). Data analyses were mainly carried out by the R (Version 4.1.0, https://www.r-project.org/) or python (Version 3.6.13, https://www.python.org/) script.

## 3 Results

### 3.1 Unifying public ARG databases for enhanced PLM-ARG training

**Figure 2.**
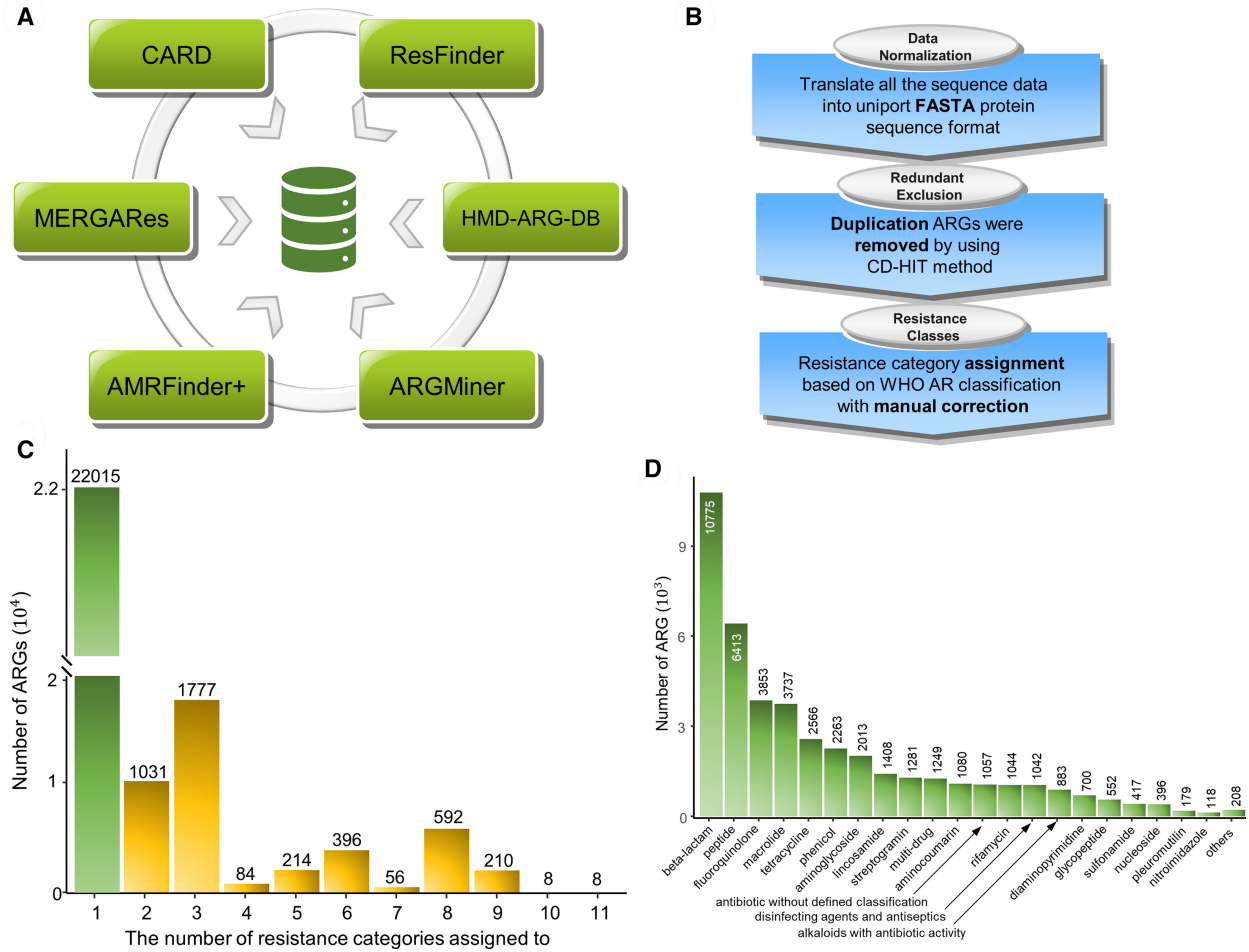
Workflow of ARG data curation and the composition of PLM-ARG database: (A) six publicly available ARG databases; (B) the proposed workflow for ARG data curation. (C) Number of ARGs with various resistance categories. (D) Distribution of ARGs across different resistance categories.

We developed a reproducible data curation schema for a benchmark ARG and resistance category classification dataset, including data normalization, redundant exclusion, and resistance class assignment (see Supplementary Materials, Fig. 2A and B). Following the proposed ARG data curation schema, we obtained a benchmark dataset for ARG and resistance category classification (i.e. PLM-ARGDB), consisting of 28 579 ARGs, including 26 391 ARGs labeled with 27 explicit resistance categories plus 2188 ARGs tagged with the fuzzy category “multi-drug” or “antibiotic without defined classification.” Specifically, PLM-ARGDB contains 4790 ARGs from CARD (i.e. 100% of proteins in CARD), 859 ARGs from ResFinder (27.94% of proteins in ResFinder), 2044 ARGs from MEGARes (30.01% of proteins in MEGARes), 444 ARGs from AMRFinderPlus (7.43% of proteins in AMRFinderPlus), 9863 ARGs from ARGMiner (66.34% of proteins in ARGMiner), and 10 579 ARGs from HMD-ARG-DB (61.30% of proteins in HMD-ARG-DB). Excluding the ARG with fuzzy category, we observed that most ARGs (22 015, 77.03%) confer resistance to only one antibiotic category. In contrast, approximately a quarter of ARGs (4376, 15.31%) resist multiple antibiotic categories (Fig. 2C). We also found that ARG distribution in different antibiotic resistance categories was extremely unbalanced. For example, of 29 antibiotic resistance categories, the top 10 resistance categories occupied approximately 82.24% of the ARGs. The top 3 resistance categories, such as beta-lactam (24.92% of ARGs), peptide (14.83% of ARGs), and fluoroquinolone (8.91% of ARGs), cover nearly half of the ARGs (Fig. 2D).

### 3.2 Comparative analysis against state-of-the-art ARG prediction methods

To demonstrate the superiority of the proposed PLM-ARG model, we compared our PLM-ARG model to three state-of-the-art ARG prediction approaches, including RGI (a sequence alignment-based method suggested by both CARD and MEGARes, version 5.2.0), Resfams (an HMM-based system, version 1.2), and DeepARG (a deep-learning-based algorithm, version v2) by using a 5-fold cross-validation method.

**Figure 3.**
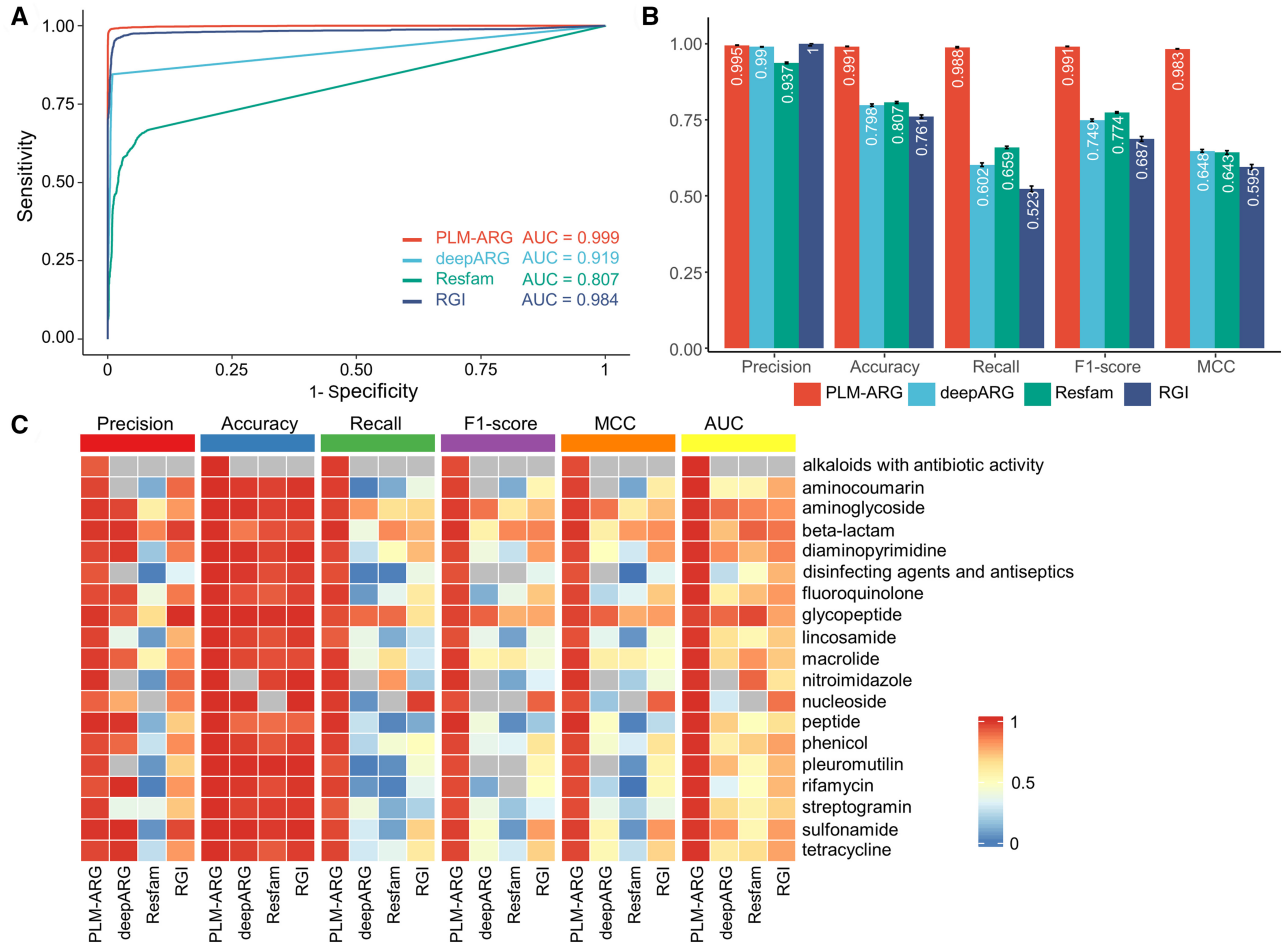
Comparison of the PLM-ARG with three state-of-the-art approaches on ARG prediction based on a 5-fold cross-validation strategy: (A) ROC curves of PLM-ARG, DeepARG, Resfams, and RGI based on 5-fold cross-validation results. (B) Performance metrics of the PLM-ARG model and three state-of-the-art approaches, including DeepARG, Resfams, and RGI, based on the 5-fold cross-validation results. (C) Performance comparison of PLM-ARG, DeepARG, Resfams, and RGI on each antibiotic-resistant category. The color bar represents the range of values for each metric, with 0 denoting the poorest performance and 1 indicating the highest or best performance. The probability was used to generate the ROC curves for PLM-ARG and DeepARG models, and the e-value was used for the Resfams model. For the RGI model, we used the ratio of Best_Hit_Bitscore to Pass_Bitscore to generate the ROC curve.

For ARG classification, we found the ranking order on the area under the ROC curve (AUROC) in a sequence of PLM-ARG > RGI > DeepARG > Resfams (Fig. 3A). The AUROC of PLM-ARG (0.999 ± 0.0003) is higher than that of RGI (0.984 ± 0.001), DeepARG (0.917 ± 0.002), and Resfams (0.807 ± 0.003). Also, PLM-ARG outperformed the other three approaches on four performance metrics (i.e. Recall, Accuracy, F1-score, and MCC) except Precision (Fig. 3B). For example, PLM-ARG yielded the highest MCC (0.983 ± 0.001), providing improvement of 51.7%, 52.9%, and 65.2% compared to DeepARG (0.648 ± 0.005), Resfams (0.643 ± 0.006) and RGI (0.695 ± 0.008), respectively. The low MCC of DeepARG and RGI may be due to the strict default thresholds. Furthermore, PLM-ARG yielded the most balanced precision (0.995 ± 0.001) and recall (0.988 ± 0.002), suggesting its superior ability to eliminate false positives and negatives. Although the DeepARG and RGI achieved the highest precision (1.000), they sacrificed recall values (i.e. 0.685 ± 0.005 for DeepARG and 0.488 ± 0.003 for RGI).

For ARG resistance category classification, PLM-ARG outperformed the other three ARG prediction methodologies concerning all the performance metrics and almost all the resistance categories (Fig. 3C). For example, PLM-ARG achieved a macro-average AUC of (0.989 ± 0.008), improving 57.74%, 48.28%, and 29.96% over DeepARG (0.627 ± 0.19), Resfams (0.667 ± 0.17) and RGI (0.761 ± 0.07), respectively. We also observed that all four methods performed well (AUC ≥ 0.8) for resistance categories with a larger number of referred ARGs, such as beta-lactam, aminoglycoside, and glycopeptide. Significantly, PLM-ARG demonstrated significantly improved performance compared to the three state-of-the-art approaches when dealing with resistance categories involving a small number of referenced ARGs. Moreover, we also computed the confusion matrix to evaluate the performance of PLM-ARG for each resistance category (Supplementary Table S1). The results showed that PLM-ARG achieved a high kappa score, a commonly used metric for evaluating classification model performance, for all resistance categories with >100 training sequences.

**Figure 4.**
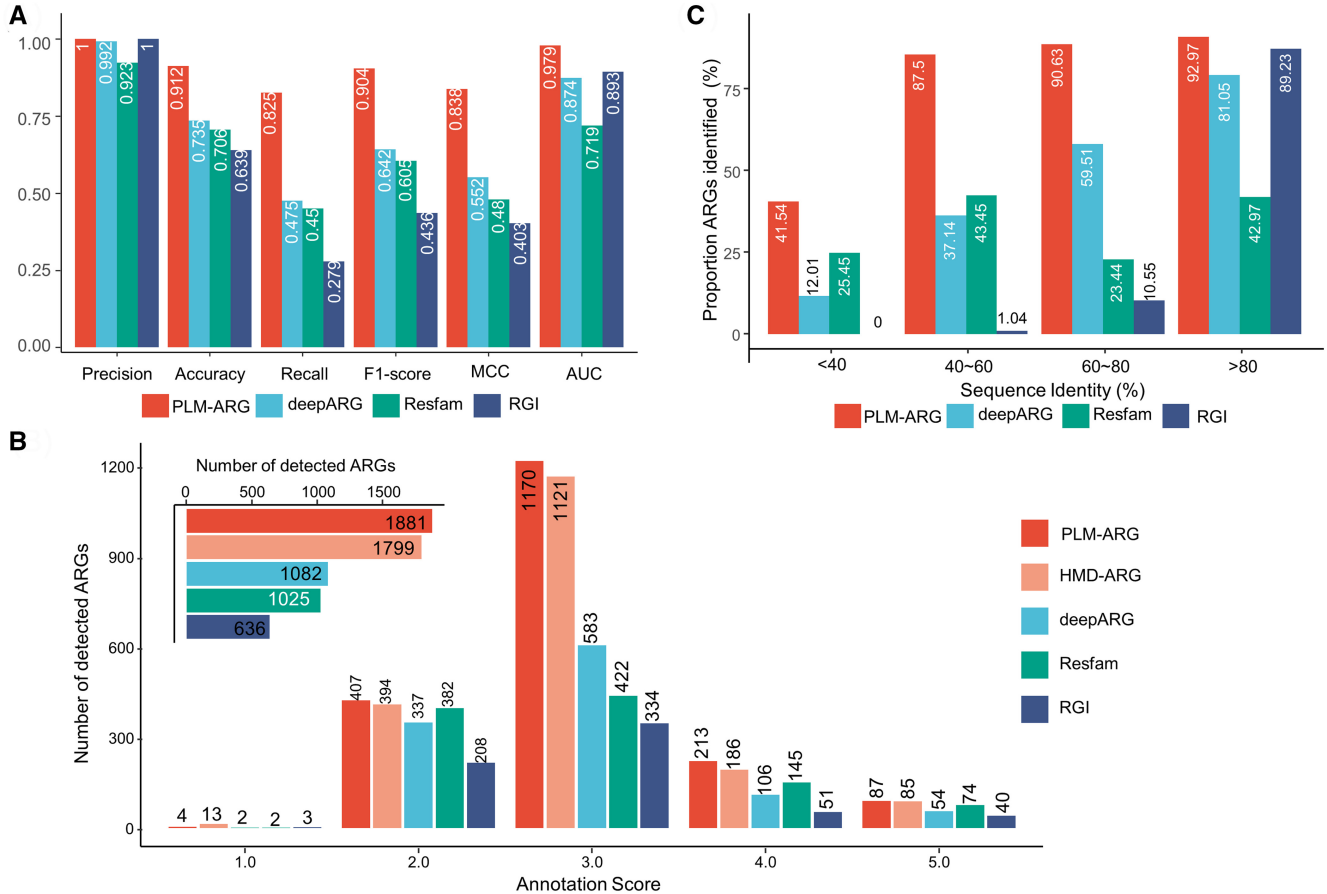
Comparison of the PLM-ARG with three the-state-of-art approaches on ARG prediction based on independent validation set: (A) performance metrics of the PLM-ARG model and three-state-of-art approaches, including DeepARG, RGI, and Resfams based on the independent validation set. (B) Number of UniProt-reviewed ARGs with different annotation scores detected by different methods. The subplot indicated the total number of UniProt-reviewed ARG detected by different methods. (C) Comparison of the ARG identification ability of the PLM-ARG model and the other three approaches on ARGs with different identities to the reference ARGs.

We also validate the performance of the developed PLM-ARG model on an independent validation set. We first retrieved bacterial ARGs protein sequences with the query “taxonomy: ‘Bacteria [2]’ AND keyword: ‘Antibiotic resistance [KW-0046]’ AND reviewed: yes” against the UniProt database, and then excluded the overlapped ARGs in the PLM-ARGDB, obtaining 2280 ARGs (released on 27 May 2022). Following the strategy outlined in the ARG data curation section, we also curated an equal number of non-ARGs that did not overlap with those in the PLM-ARGDB. As a result, an independent validation consisting of an equal number (i.e. 2280) of ARGs and non-ARGs was constructed to evaluate the PLM-ARG model. Due to the lack of ground truth in resistance category information, the comparison focused on the ARG identification. We observed the same ranking order on all the performance metrics except precision as those of the 5-fold cross-validation, i.e. PLM-ARG > RGI > DeepARG > Resfams (Fig. 4A). With notable significance, PLM-ARG has achieved an impressive MCC of 0.838, signifying a substantial advancement over alternative methodologies within the range of 51.8%–107.9%. Moreover, PLM-ARG demonstrates exceptional prowess in the F1-score metric, boasting a value of 0.904, indicative of a noteworthy improvement compared to alternative methods spanning from 40.8% to 107.3%. In addition, PLM-ARG secures the highest AUC value, reaching 0.979 and manifesting an improvement ranging from 9.6% to 36% when juxtaposed against three prominent state-of-the-art approaches for ARG prediction.

Furthermore, we assessed the distribution of annotation quality scores among UniProt-reviewed ARGs predicted by various methods (see Section 2), utilizing the annotation quality score supplied by the UniProt database. In this regard, we included the HMD-ARG method, which provides a web server for ARG prediction, in our analysis. Our findings demonstrate that PLM-ARG could detect more UniProt-reviewed ARGs than other methods, particularly for ARGs with high annotation scores (Fig. 4B). These results further affirm the high reliability of PLM-ARG.

To investigate whether the proposed PLM-ARG could have better discrimination power for ARGs that are not similar to the ones in the training set, we divided the 2280 UniProt-reviewed ARG in the independent test set into 5 groups according to its similarity with the referenced ARGs in the training set. We summarized the individual rate of the proportion of ARGs identified in the four methodologies. The results showed that PLM-ARG could identify >80% of the ARGs in different similarity groups except for those with <20% similarity (Fig. 4C). RGI can hardly identify ARG with <60% similarity, although its precision is the highest. The low performance of Resfam for the ARGs with high identity may be due to the lack of available HMM models for the reference ARGs.

Moreover, to investigate the decline in performance across all methods within the subset of ARGs sharing <40% identity with the referenced ARGs, we conducted a BLAST analysis of these low-identity ARGs against the non-ARGs present in the training dataset. Our analysis revealed that 35.5% of these ARGs exhibited higher similarity to the non-ARGs present in the training dataset. Notably, our proposed methodology successfully identified 41.54% of these ARGs, surpassing the performance of other contemporary state-of-the-art approaches.

### 3.3 Case study: resistance category annotation of the unreviewed ARGs in UniProt

**Figure 5.**
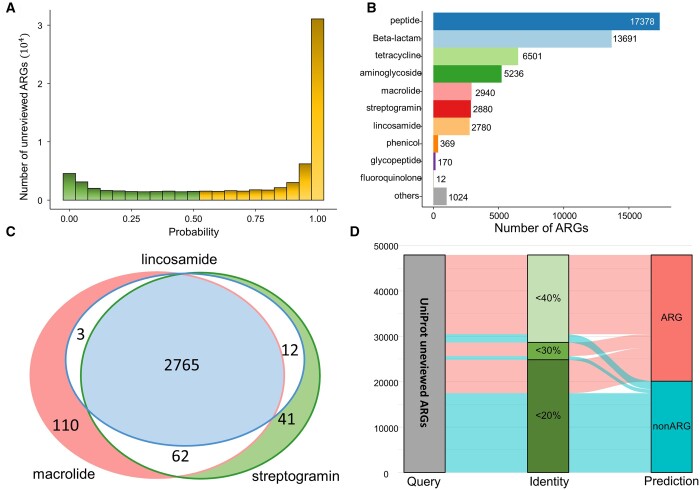
Resistance category annotation of the unreviewed ARGs in UniProt using PLM-ARG: (A) distribution of ARG across different prediction probabilistic value bins; (B) predicted resistance category of the unreviewed ARGs in UniProt; (C) overlap of ARGs conferring resistance to macrolides, lincosamides, and streptogramines, and (D) percentage of unreviewed ARGs with extremely low similarity to the referenced ARGs identified by the proposed PLM-ARG.

A substantial portion of unreviewed bacterial proteins in the UniProt database were categorized as antibiotic resistance, yet lacked specific detailed information about resistance categories. To fill the gap, we utilized our developed PLM-ARG to prioritize unreviewed ARGs in the UNIPROT database and expand their resistance category annotations (see Supplementary Materials). Of 73 938 unreviewed ARGs, PLM-ARG predicted respective 38 996 (52.74%) and 13 902 (18.8%) with probabilistic value intervals 0.9–1 and 0.5–0.9 as ARGs (Fig. 5A). Among these identified 52 898 ARGs (probabilistic value > 0.5), 46 343 ARGs (87.61%) can be assigned with explicit resistance categories. The top three assigned resistance categories were peptide, beta-lactam, and tetracycline (Fig. 5B). Most of these identified ARGs (93.84%) were conferred only one class of antibiotics. As a typical exception, the antibacterial spectre and mechanisms of macrolides, lincosamides, and streptogramines (MLS) are similar. The genes conferring resistance to one of three classes of antibiotics are also conferring resistance to the other two classes of antibiotics. This phenomenon was also observed in our prediction results (Fig. 5C). We can see that 2993 genes were assigned with at least one of the MLS categories, and 92.38% conferred resistance to all three categories of antibiotics. The specific resistance categories assigned to the 73 938 unreviewed ARGs, along with their predicted probabilistic values, have been provided in Supplementary Table S2. This resource is intended to facilitate further assessment and prioritization by the scientific community.

Furthermore, we calculated the identity between the 73 938 unreviewed ARGs and our PLM-ARGDB and found 64.77% of them with a similarity of <40%, which were defined as distant homologous ARGs. We found that 58% of these distant homologous ARGs could be predicted with PLM-ARG. Notably, 29.67% of the ARGs with <20% similarity can also be identified. These results further demonstrated the strong ability of PLM-ARG for ARG identification (Fig. 5D). The category classification also had high reliability. For example, the UniProt protein A0A010ZRL4, with <20% similarity to the reference ARGs, was predicted as tetracycline. Its function is recorded as the repressor of the tetracycline resistance element in the UniProt database. Besides that, the UniProt protein A0A023CZL5, with <20% similarity to the reference ARGs, was predicted as a peptide. Its functions are recorded to be involved in the resistance mechanism against cationic antimicrobial peptides.

In addition to augmenting resistance annotation within the UniProt database, we also demonstrated the practical applicability of the PLM-ARG framework in investigating the diversity of the resistome within Earth's environmental microbiota. This highlights its substantial potential for real-world applications (see Supplementary Materials).

## 4 Discussion and conclusion

Environmental and commensal bacteria harbor a diverse and largely unknown collection of ARGs that may be mobilized and transferred to pathogens, impacting public health over time. Computational approaches using the accumulated omics data offer an effective way for ARG identification and resistance category annotation. Here, we developed a novel Artificial Intelligence (AI)-powered language model framework, PLM-ARG, for ARG and resistance category classification by translating sequence data to protein language. The proposed method outperformed the state-of-the-art approaches with a 9.6%–36% range of improvement over AUC and 40.8%–107.3% over the F1-score based on the independent validation set. Although we demonstrated the superiority of the PLM-ARG on distant homologous ARGs by comparing it with three popular ARG prediction approaches (i.e. RGI, Resfams, and DeepARG), we have come to recognize that there is no single method that can cater to all real-world applications. Therefore, the pivotal consideration in choosing a “fit-for-purpose” strategy is to meticulously align it with the particular context of the real-world application.

It is widely acknowledged that bacteria can develop resistance to specific antibiotics by acquiring ARGs and through point mutations in chromosomal target genes associated with antibiotic resistance. Although this study only focuses on acquired genes, it is crucial to acknowledge that resistance caused by point mutations in genes differs significantly from acquired genes. Thus, identifying resistance caused by point mutations is vital in comprehending the mechanism of bacterial resistance and should receive more attention in future research. In addition, defining resistance categories more precisely would be beneficial, such as grouping beta-lactams into different sub-classes, enhancing their clinical applications.

Additional investigations are worth considering to improve the model performance of PLM-ARG further and confirm the findings from this study: (i) PLM-ARG takes the assembled genes/ORF as the input, hampering its identification of low-abundance ARGs that cannot be assembled without enough sequencing depth. Therefore, further investigation on directly modeling the sequence data (e.g. pair-end short-reads sequencing and Nanopore sequencing) may be more desirable and might hold more potential for further improving the performance of ARG identification. (ii) Due to the current constraints in our knowledge of ARGs, we cannot assure a 100% certainty regarding the selection of genes as negative controls or the comprehensiveness of assigned resistance categories for ARGs. Therefore, it is imperative to use a robust strategy when constructing the training dataset to enhance the reliability of most ARG identification methods. (iii) Given the substantial imbalance in the number of genes resistant to various antibiotic categories, there is a risk of performance deterioration, particularly for those resistance categories with fewer protein sequences. Therefore, we recommend adopting an appropriate loss function or preprocessing strategy to rebalance the class prevalence. (iv) In the current study, we showed the great potential of an AI-powered language model for ARG identification. As it needs a long computation time to generate the embedding representation for a protein sequence by the ESM-1b model, which limits the application, a lightweight model is necessary for further improvement (Liu *et al.* 2021).

In summary, PLM-ARG offers a robust, one-stop solution for both ARG identification and resistance category classification. Its independence from sequence similarity makes PLM-ARG well-suited for characterizing antibiotic resistance diversity in large-scale microbiome studies.

## Supplementary Material

btad690_Supplementary_Data

## Data Availability

The data underlying this article are available at https://github.com/Junwu302/PLM-ARG.
