# Supplementary material for: PLM-ARG: antibiotic resistance gene identification using a pretrained protein language model
Source: Bioinformatics. 2023 Nov 23;39(11):btad690. doi: 10.1093/bioinformatics/btad690 (PMC10676515; doi:10.1093/bioinformatics/btad690)
Supplement: btad690_Supplementary_Data [file btad690_supplementary_data.zip › supplementary materials.docx]

## Supplementary Materials

1. **PLM-ARG database construction**

To create a benchmark ARG data for PLM-ARG model development, we incorporated and standardized the curated AGRs from five databases, including CARD (Release Date: 05/27/2022) (Alcock, et al., 2020), ResFinder (Release Date: 24/05/2021)(Kleinheinz, et al., 2014), MEGARes (Release Date: 14/10/2019) (Doster, et al., 2020; Lakin, et al., 2017), ARGMiner (Release Version: v1.1.1.A) (Arango-Argoty, et al., 2020), AMRFinderPlus (Release Date: 11/08/2021)(Feldgarden, et al., 2019) and HMD-ARG-DB(Li, et al., 2021). First, given that various genomic technologies identified the ARGs in these databases, we converted all ARGs with DNA sequence format into UniProt FASTA protein sequence format using EMBOSS tool Transeq (Rice, et al., 2000). We accorded priority to the ARGs from different databases according to the confidence of the databases (CARD > ResFinder > MEGARes > AMRFinderPlus > ARGMiner > HMD-ARG-DB) and then clustered them with CD-HIT [37], and removed the duplicates with 100% identity and the same length were discarded. Finally, resistance categories of ARGs were assigned based on their conferred antibiotic drug classes with manual correction based on the WHO access, watch, reserve, classification of antibiotics for evaluation and monitoring of use (AWaRe) classification system (<https://apps.who.int/iris/rest/bitstreams/1374989/retrieve>), which provides official classification for a list of 257 antibiotics. As a result, a total of 28,597 ARGs, including 26,391 ARGs labeled with 27 explicit resistance categories plus 2,188 ARGs labeled with either fuzzy category "multi-drug" or "antibiotic without defined classification", were obtained.

To curate a negative data set (i.e., non-ARGs) for the PLM-ARG model development, we retrieved non-ARGs from the UniProt database with the query *"taxonomy: "Bacteria [2]" NOT keyword: "Antibiotic resistance [KW-0046]" AND "reviewed status: yes"*, yielding 333,280 potential non-ARGs (<https://www.uniprot.org/>, Release Date: 27/05/2022). To keep balanced positive and negative samples, we randomly selected 28,597 non-ARGs as the negative set for the model development. The complete curated ARGs were stored and accessible through the PLM-ARG database (i.e., PLM-ARGDB).

1. **PLM-ARG application**

To navigate the utility of the proposed PLM-ARG framework, we showcased two applications, including resistance category annotation and exploration of the resistance diversity across various environmental conditions.

**2.1 Resistance category annotation for Uniprot ARGs**

Uncovering the ARG resistance categories using wet-lab approaches is very time-consuming and labor-intensive. Sequence atlases such as UniProt contain a wealth of ARG genes. However, the detailed ARG-associated resistance category information is missing. The proposed PLM-ARG could be an effective tool for resistance category annotation. To explore the potential of the proposed PLM-ARG for resistance category annotation expansion, we utilized the PLM-ARG model to predict the resistance categories of unreviewed ARGs in the UniProt database. We retrieved unreviewed ARGs from the UniProt database by querying "taxonomy: "Bacteria [2]" AND keyword: "Antibiotic resistance [KW-0046]" AND reviewed: no". Consequently, a total of 203,165 unreviewed bacterial genes were labeled as antibiotic resistance (i.e., unreviewed ARGs). Then, we removed the redundant proteins using CD-HIT with default parameters, resulting in 73,938 non-redundant unreviewed ARGs.

**2.2 Exploration of the ARG resistance diversity in the environment**

Understanding the environment as a source of and dissemination route for ARGs is crucial in identifying potential risk scenarios for human health. Uncovering the distribution and association of ARGs across diverse environmental conditions has enormous potential to increase our understanding of the spread and persistence of ARG and promote the concept of 'one health'. The Earth’s Microbiomes (GEM) catalog collected more than 10,000 metagenomes from diverse habitats from all the Earth’s continents and oceans, engineered environments, human and animal hosts, to natural and agricultural soils (Nayfach, et al., 2021). To further demonstrate the utility of PLM-ARG and explore the resistome diversity of various earth’s environmental microbiota, we randomly selected 100 samples of seven different environments (e.g., human gut, skin, building, wastewater, marine, and freshwater). We then employed our developed PLM-ARG model to identify potential ARGs from the 52,515 MAGs and conducted a diversity analysis of ARG across different environmental conditions. The proportion of ARGs in all the prediction genes was used to profile the resistome for each sample. We calculated the Shannon index to measure the diversity of the resistance category in specific ecological conditions (Tucker, et al., 2017). For each sample, the Shannon diversity index could be calculated based on the following equation:

$$\begin{aligned} H=-\sum p_{i}*\ln\left( p_{i} \right) \#\left( 6 \right) \end{aligned}$$

where $p_{i}$ is the proportion of the entire ARG set made up of resistance category $i$. The higher the value of $H$, the higher the diversity of resistance category in the sample.

**2.3 PLM-ARG development**

The proposed PLM-ARG framework employed the XGBoost algorithm for ARG prediction and resistance category classification based on protein representation generated from the pre-trained ESM-1b protein language model. "In order to underscore the protein language model's prowess in ARG classification, we conducted a dimensionality reduction analysis utilizing UMAP, projecting the dataset of 28,597 ARGs and non-ARGs into a two-dimensional space (Figure S1A). The outcomes of this visualization reveal a compelling trend: ARGs belonging to diverse resistance categories exhibit a tendency to cluster together, thus signifying the discriminative capability inherent in the protein representations derived from the ESM-1b model.

The hyperparameters of XGBoost model Five hyperparameters of the XGBoost model, specifically *n_estimators*, *max_depth*, *learning_rate*, *subsample*, and *colsample_bytree,* were meticulously optimized using the GridSearchCV procedure. The search intervals for them were thoughtfully defined as follows: [80, 100, 120, 140, 160, 180, 200, 220, 240, 260, 280, 300] for *n_estimators*, [3, 4, 5, 6, 7, 8, 9] for *max_depth*, [0.01, 0.1, 0.2, 0.3] for *learning_rate*, [0.5, 0.6, 0.7, 0.8, 0.9, 1] for *subsample*, and [0.5, 0.6, 0.7, 0.8, 0.9, 1] for *colsample_bytree*. A total of 12,096 XGBoost models were further developed based on generated protein representations under five hyperparameter combinations (i.e., subsample, colsample_bytree, learning_rate, max_depth, and n_estimators). In general, the average value and standard deviation of AUCs yielded from the 12,096 XGBoost models were > 0.97±1.4e-3, suggesting the high predictive power of the XGBoost algorithm for ARG identification. Specifically, there was no statistical significance among AUCs from the XGBoost models with different *subsample* and *colsample_bytree* settings (Figure S1B). The XGBoost model yielded the highest average AUC and lowest standard deviation (avg_auc=0.998±6.7e-4, std = 2.5e-4±4.8e-5) at *learning_rate*=0.1 (Figure S1C). The average AUC values tended to be stable after the *max_depth* of 6, while the smallest standard deviation of AUCs reached at the *max_depth* of 7 (Figure S1D). Similarly, the average AUC in different n_estimators > 200 settings were all more than 0.9995, while the smallest standard deviation of AUCs was achieved when the *n_estimators* was set as 200 (Figure S1E). Therefore, we employed the XGBoost model with hyperparameter setting, including subsample and *colsample_bytree* as default values, *learing_rate*=0.1, *max_depth*=7, and *n_estimators*=200, as the PLM-ARG model for further analysis.

Moreover, with a focus on achieving a balance between high performance and computational efficiency, we conducted an in-depth examination of various sequence trimming lengths (100, 200, 400, 600, 800, and 1000) through 5-fold cross-validation. Our analysis revealed that PLM-ARG's performance exhibited robust stability when the trimming length equaled or exceeded 200 amino acids. Consequently, we adopted a trim length of 200 amino acids for our study (Figure S1F).


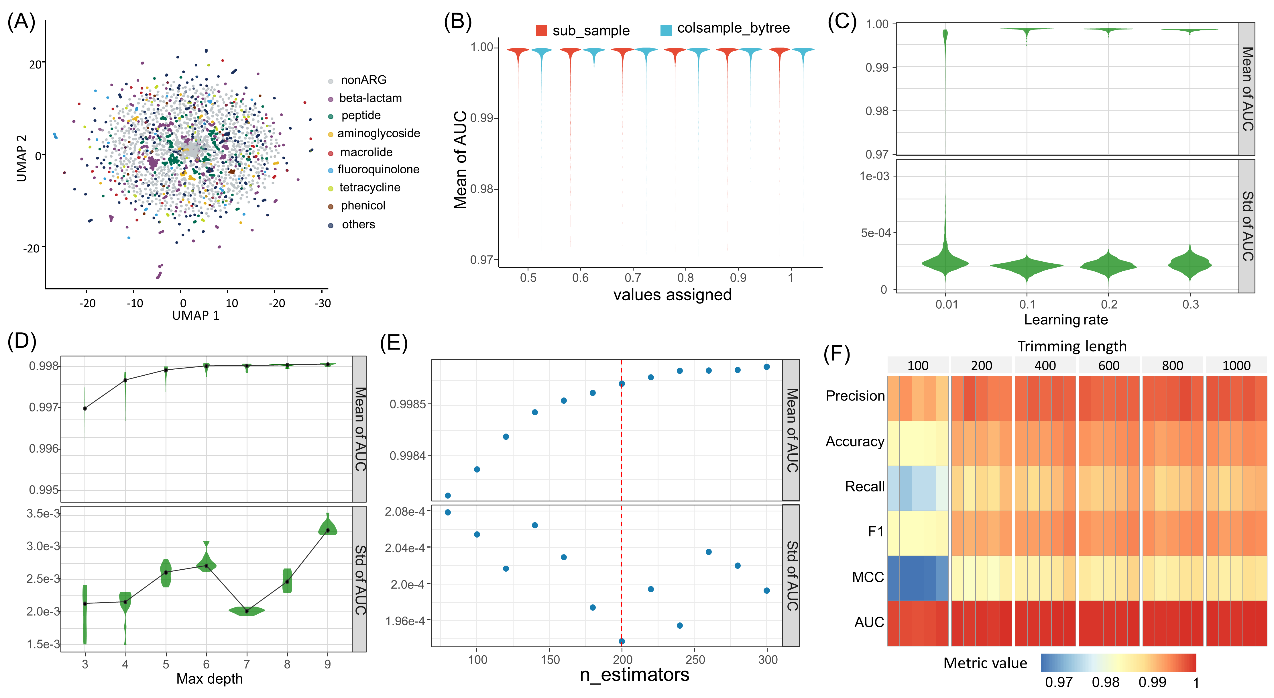


**Figure S1 Hyperparameter optimization for the PLM-ARG development.** **A)** UMAP projection (McInnes, et al., 2018) of protein representation of 28,597 ARGs generated by ESM-1b model, colors correspond to the resistance categories. **B)** Distribution of mean and standard deviations of AUCs of XGBoost models with different *sub_sample* and *colsample_bytree*. **C-E)** Distributions of mean and standard deviations of AUCs of XGBoost models with different *learning rates*, *max_depth*, and *n_estimators*. **F)** The 5-fold cross-validation results of the PLM-ARG model with varied input protein trimming lengths.

1. **Case study - Exploring resistome diversity of the Earth's environmental microbiota**

The extensive use of antibiotics leading to the rapid spread of antibiotic resistance poses high health risks to humans, but little is known about the environmental reservoirs of resistance genes. To depict the resistome of Earth's environmental microbiota, we applied PLM-ARG to identify potential ARGs and their resistance categories from the 52,515 metagenome-assembled genomes (MAGs) of Earth's Microbiomes (GEM) (release date: 30/11/2020) and investigate their association with different environmental conditions (Nayfach, et al., 2021).

To demonstrate the utility of the proposed PLM-ARG model, we randomly selected 100 samples related to each of the seven environments, including the human gut, wastewater, building, soil, marine, and freshwater, recorded in the GEM databases. Then we predicted the ARGs in each sample (Supplementary Table S3). The results showed that the building samples harbored the highest proportion of ARGs, followed by the skin and human gut samples (Figure S2A). Similarly, building samples' resistome diversity was the highest, followed by human gut and wastewater samples. The proportion of ARGs in different resistance categories across different environments were shown in Figure S2B. We observed that the proportion of ARGs conferring resistance to beta-lactam antibiotics was highest in all the environments. More specifically, the building samples harbored the highest proportion of ARGs conferring resistance to most categories of antibiotics, including beta-lactm, aminoglycoside, peptide, tetracycline, fluoroquinolone, and aminocoumarin, indicating that antibiotic resistance situation of buildings needs more attention. We also observed that the proportion of ARG resistant to glycopeptide, streptogramin, and macrolide is highest in the human gut samples. Glycopeptides and streptogramins are potent drugs against numerous highly resistant pathogens and are considered as antibiotics of last resort in human therapy (Donadio and Sosio, 2009; Mast and Wohlleben, 2014). The high proportion of ARGs conferring resistance to glycopeptide further indicated the emergency and severity of ARG spreading to human health. Besides that, the skin samples were also observed harboring the highest proportion of ARGs conferring resistance to lincosamide, which was used to treat skin and soft tissue infections.

We also investigated the beta diversity of the antibiotic resistome across different environmental samples. We found that the samples obtained from different environments were significantly separated (Adonis test, R2=0.31 and p = 0.01) based on the Bray-Curtis distance (Figure S2C). We further investigated a homogenization effect of the environment on the ARG composition. We used the betadisper R function(Oksanen, et al., 2019) to calculate the dispersion within each environment based on a Bray-Curtis dissimilarity matrix. Then ANOVA was conducted to test the significant differences in beta diversity among different environmental samples (Figure S2D, betadisper ANOVA F, = 21.26, p = 5.69e-23). The results showed that soil samples were the most variable in ARG composition. In contrast, the human gut and wastewater samples variable was less than those obtained from other environments.


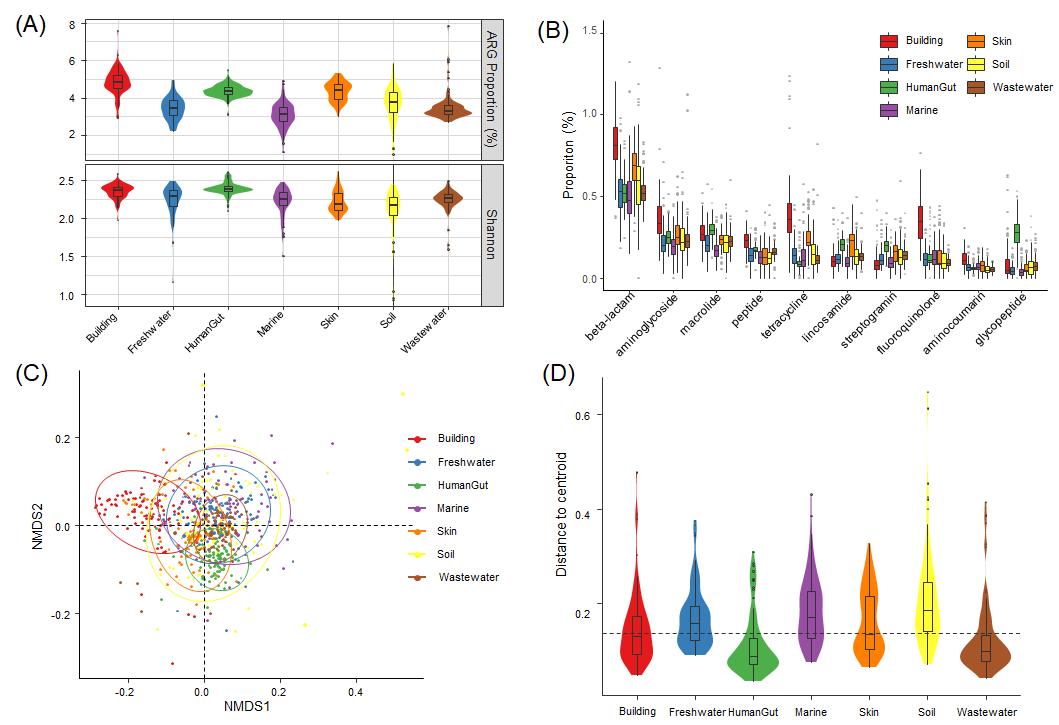


**Figure S2**. The resistome diversity of Earth's environmental microbiota: **A)** The proportion of ARGs and resistome diversity of the samples collected from different environments. **B)** Proportion of ARGs conferring different resistance categories of antibiotics across different environments. **C)** Nonmetric multidimensional scaling (NMDS) plot of Bray-Curtis dissimilarities between resistome of samples obtained from different environments. **D)** Dispersion among resistome from different environments. Greater distances to the centroid (y-axis) indicate more variability in ARG composition.

1. **Reference**

Alcock, B.P., et al. CARD 2020: antibiotic resistome surveillance with the comprehensive antibiotic resistance database. Nucleic Acids Res 2020;48(D1):D517-D525.

Arango-Argoty, G.A., et al. ARGminer: a web platform for the crowdsourcing-based curation of antibiotic resistance genes. Bioinformatics 2020;36(9):2966-2973.

Donadio, S. and Sosio, M. Glycopeptides, Antimicrobial. In: Schaechter, M., editor, Encyclopedia of Microbiology (Third Edition). Oxford: Academic Press; 2009. p. 455-471.

Doster, E., et al. MEGARes 2.0: a database for classification of antimicrobial drug, biocide and metal resistance determinants in metagenomic sequence data. Nucleic Acids Res 2020;48(D1):D561-D569.

Feldgarden, M., et al. Validating the AMRFinder tool and resistance gene database by using antimicrobial resistance genotype-phenotype correlations in a collection of isolates. 2019;63(11):e00483-00419.

Kleinheinz, K.A., Joensen, K.G. and Larsen, M.V. Applying the ResFinder and VirulenceFinder web-services for easy identification of acquired antibiotic resistance and E. coli virulence genes in bacteriophage and prophage nucleotide sequences. Bacteriophage 2014;4(1):e27943.

Lakin, S.M., et al. MEGARes: an antimicrobial resistance database for high throughput sequencing. Nucleic Acids Research 2017;45(D1):D574-D580.

Li, Y., et al. HMD-ARG: hierarchical multi-task deep learning for annotating antibiotic resistance genes. Microbiome 2021;9(1).

Mast, Y. and Wohlleben, W. Streptogramins–two are better than one! Int J Med Microbiol 2014;304(1):44-50.

McInnes, L., Healy, J. and Melville, J. Umap: Uniform manifold approximation and projection for dimension reduction. arXiv preprint arXiv:1802.03426 2018.

Nayfach, S., et al. A genomic catalog of Earth’s microbiomes. Nature Biotechnology 2021;39(4):499-509.

Nayfach, S., et al. A genomic catalog of Earth's microbiomes. Nat Biotechnol 2021;39(4):499-509.

Oksanen, J., et al. Vegan: community ecology package (version 2.5-6). The Comprehensive R Archive Network 2019.

Rice, P., Longden, I. and Bleasby, A. EMBOSS: The European molecular biology open software suite. Trends Genet 2000;16(6):276-277.

Tucker, C.M., et al. A guide to phylogenetic metrics for conservation, community ecology and macroecology. Biol Rev Camb Philos Soc 2017;92(2):698-715.
