# Supplementary figures and images for: PLM-ARG: antibiotic resistance gene identification using a pretrained protein language model
Source: Bioinformatics. 2023 Nov 23;39(11):btad690. doi: 10.1093/bioinformatics/btad690 (PMC10676515; doi:10.1093/bioinformatics/btad690)

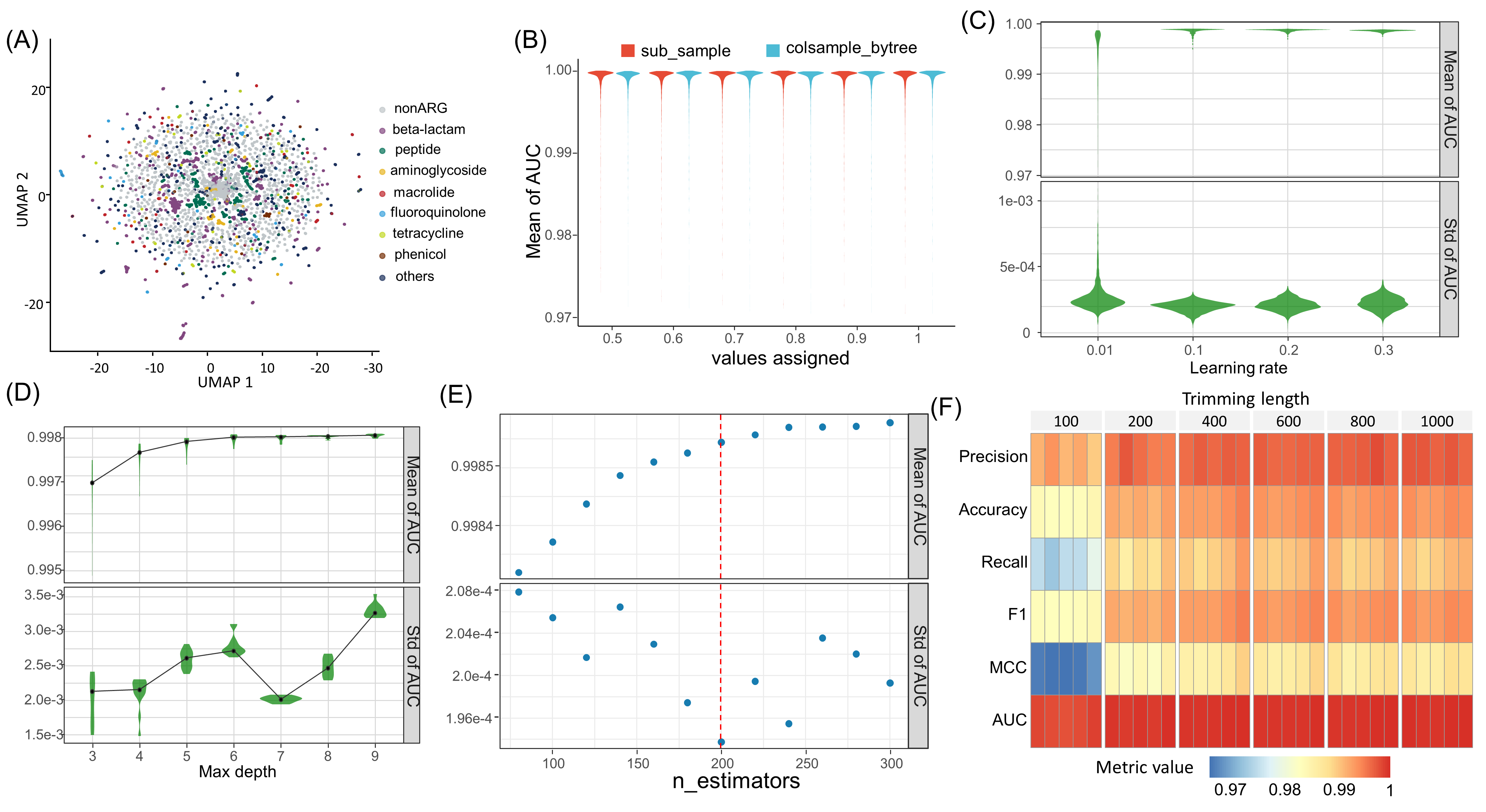

Supplement: btad690_Supplementary_Data [file btad690_supplementary_data.zip › Fig S1.tif]

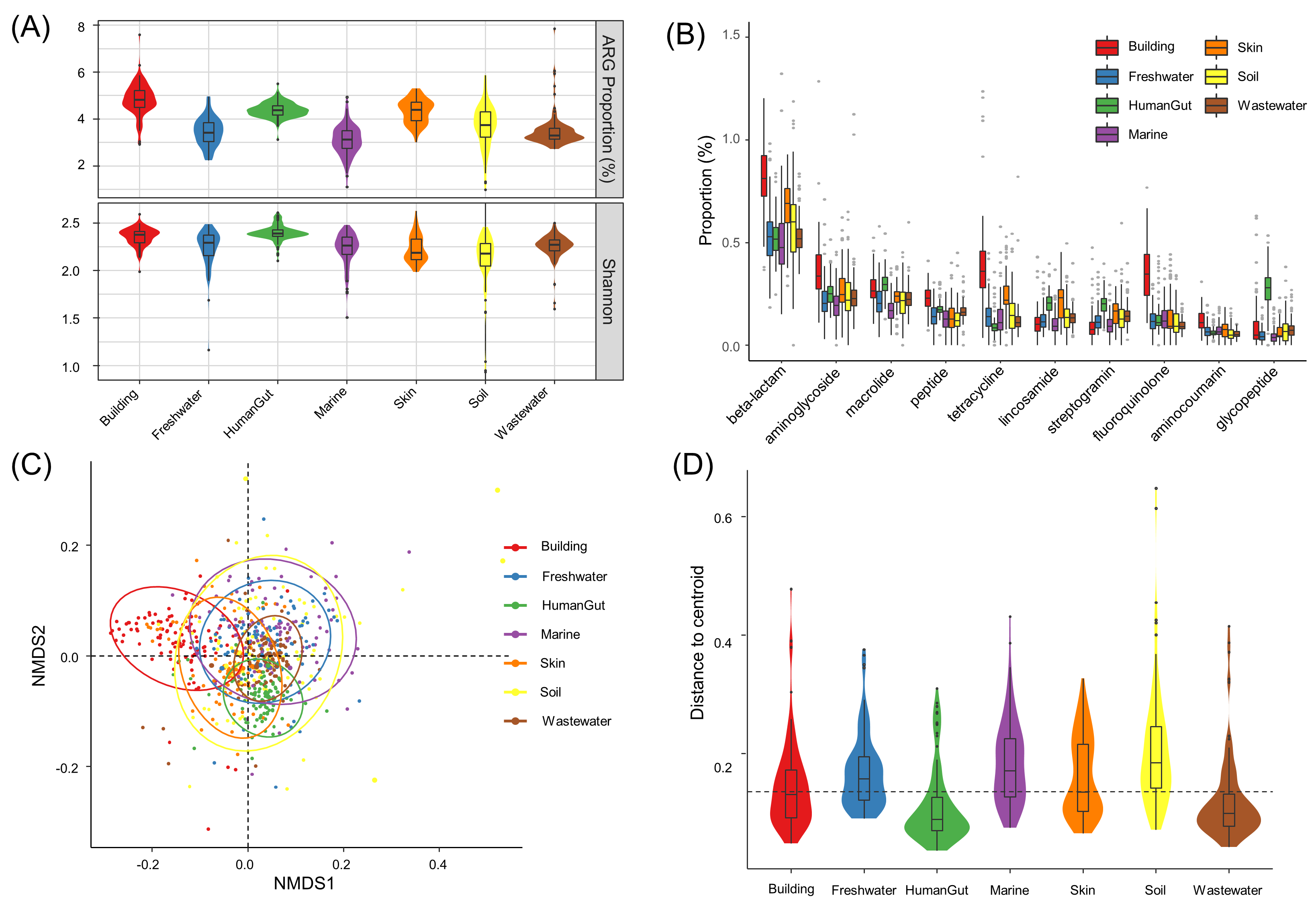

Supplement: btad690_Supplementary_Data [file btad690_supplementary_data.zip › Fig S2.tif]
